# GC-MS Analysis of the Volatile Constituents in the Leaves of 14 Compositae Plants

**DOI:** 10.3390/molecules23010166

**Published:** 2018-01-18

**Authors:** Yiguang Wang, Xiran Li, Qinjie Jiang, Hainan Sun, Jiafu Jiang, Sumei Chen, Zhiyong Guan, Weimin Fang, Fadi Chen

**Affiliations:** College of Horticulture, Nanjing Agricultural University, Key Laboratory of Landscape Agriculture, Ministry of Agriculture, Nanjing 210095, China; wangyiguang1990@163.com (Y.W.); 14113111@njau.edu.cn (X.L.); 2016804134@njau.edu.cn (Q.J.); sunhainan1989@hotmail.com (H.S.); jiangjiafu@njau.edu.cn (J.J.); chensm@njau.edu.cn (S.C.); guanzhy@njau.edu.cn (Z.G.); fangwm@njau.edu.cn (W.F.)

**Keywords:** Compositae plants, leaves, GC-MS, terpenoids, chemotaxonomy

## Abstract

The green organs, especially the leaves, of many Compositae plants possess characteristic aromas. To exploit the utility value of these germplasm resources, the constituents, mainly volatile compounds, in the leaves of 14 scented plant materials were qualitatively and quantitatively compared via gas chromatography-mass spectrometry (GC-MS). A total of 213 constituents were detected and tentatively identified in the leaf extracts, and terpenoids (especially monoterpene and sesquiterpene derivatives), accounting for 40.45–90.38% of the total compounds, were the main components. The quantitative results revealed diverse concentrations and compositions of the chemical constituents between species. Principal component analysis (PCA) showed that different groups of these Compositae plants were characterized by main components of α-thujone, germacrene D, eucalyptol, β-caryophyllene, and camphor, for example. On the other hand, cluster memberships corresponding to the molecular phylogenetic framework, were found by hierarchical cluster analysis (HCA) based on the terpenoid composition of the tested species. These results provide a phytochemical foundation for the use of these scented Compositae plants, and for the further study of the chemotaxonomy and differential metabolism of Compositae species.

## 1. Introduction

Emissions of volatile or aroma components from plant organs, including green tissues and flowers, play important roles in defending plants from pests, attracting pollinators, and increasing competitive advantages by inhibiting the growth of surrounding plants [1]. The green tissues of many Compositae members, which are characterized by unique aromas contain abundant volatile chemicals. An example is the genus *Artemisia*, the members of which release strong scents from their aerial parts [2]. Species in other genera, such as *Chrysanthemum indicum* var. *aromaticum*, a new variety of *Chrysanthemum indicum* endemic to Shennongjia, Hubei Province, China [3], are known for a special and tangy flavor throughout the whole plant [4]. *Opisthopappus* plants, including *Opisthopappus taihangensis* and *Opisthopappus longilobus* are also native to China, and are primarily restricted to the Taihang Mountains [5]. The volatile constituents contributing to the herbal scent were principally identified as terpenoid compounds. The terpenoids detected in the aerial parts of some *Artemisia* plants include monoterpenes, such as α-pinene, α-terpinene and camphene; sesquiterpenes, such as β-caryophyllene, germacrene D, α-copaene and α-cubebene; and oxygenated terpenes, such as eucalyptol, camphor, and thujone [2,6,7]. Many of these compounds are regarded as aroma-active because of their special odors [8].

Due to their chemical components, many of these scented plants, have been used in folk medicines around the world. Numerous *Artemisia* plants, including *Artemisia annua*, *Artemisia argyi*, *Artemisia sacrorum*, *Artemisia capillaris*, and *Artemisia japonica*, are used as traditional Chinese medicine and recorded in ancient Chinese pharmacopoeias [9]. Both *Artemisia afra* and *Artemisia herba-alba* are used to treat coughing, colds, and other diseases in Africa [10,11]. In Turkey, *Artemisia absinthium*, *Artemisia spicigera*, and *Artemisia santonicum* are local folk medicines; among these, *A. absinthium* has pharmacological functions such as antipyretic, antiseptic, anthelmintic, tonic, and diuretic activities, and *A. santonicum* acts as an anthelmintic and diabetes drug [12]. Other species, such as *C. indicum* var. *aromaticum* and *Opisthopappus* plants, also have pharmacological potential and are used to treat diseases in their origin areas [13,14]. Another famous traditional Chinese medicine, *Crossostephium chinense*, is commonly used to treat colds, rheumatism, and arthralgia [15]. *Tanacetum vulgare*, an aromatic perennial plant that grows widely in Europe, Asia and North America, is regarded as a food additive and a resource for perfumery and herbal medicine with multifunctional properties [16,17,18]. The essential oils of *T. vulgare* have been proven to possess the anti-inflammatory [19], antifungal [20] and antioxidant [21] activities.

The volatile oils extracted from Compositae plants, especially the essential oils of many *Artemisia* plants, have been demonstrated to possess insecticidal activities against grain storage pests [22,23,24,25]. Since the secondary plant metabolites have low mammalian toxicity, undergo rapid degradation, and are more environmentally benign, they are considered as effective substitutes for traditional pesticides [26]. Not limited to insects, the growth of other surrounding plants can also be affected by the constituents from at least 39 genera of Compositae plants [27]. In addition, the phytotoxic effects of different constituents acting on plants are reportedly diverse, with the effects of four monoterpenes on maize observed as camphor > eucalyptol > α-pinene > limonene [28,29]. Thus, the allelochemicals from scented plants show great promise for applications in regulating plant growth and producing natural herbicides.

Since many Compositae plants possess various volatile chemicals with high utilization value, compositional investigations and comparisons should be conducted in more species. Several methods of extracting or collecting volatile compounds exist [30]. To explore all endogenous components, i.e., not limited to the volatile components, and avoid the high-temperature destruction of certain compounds during hydrodistillation, solvent extraction followed by gas chromatography-mass spectrometry (GC-MS) was used to analyze the chemical constituents of fresh leaves from 14 Compositae plants in this research. To our knowledge, The volatile components of several of the investigated plants, such as *Opisthopappus*, *Artemisia yunnanensis*, and *Artemisia vulgaris* ‘Variegate’ (which is a cultivar of *A. vulgaris* with golden and mottled leaves) have rarely been reported previously. Additionally, many previous studies on volatile compounds have focused on qualitative analyses or differences in proportions (relative content) within a single species, and a focus on quantitative analyses is comparatively lacking. Thus, to characterize these scented plants and compare the constituents between species, qualitative and quantitative data were analyzed using several statistical methods. Furthermore, hierarchical cluster analysis (HCA) of the terpenoid compositions in these species was performed to evaluate the effectiveness of chemotaxonomy, which could be an auxiliary to phylogenetic systematics.

## 2. Results

### 2.1. Identification and Quantification of the Chemical Constituents in 14 Compositae Plants

**Figure 1:**
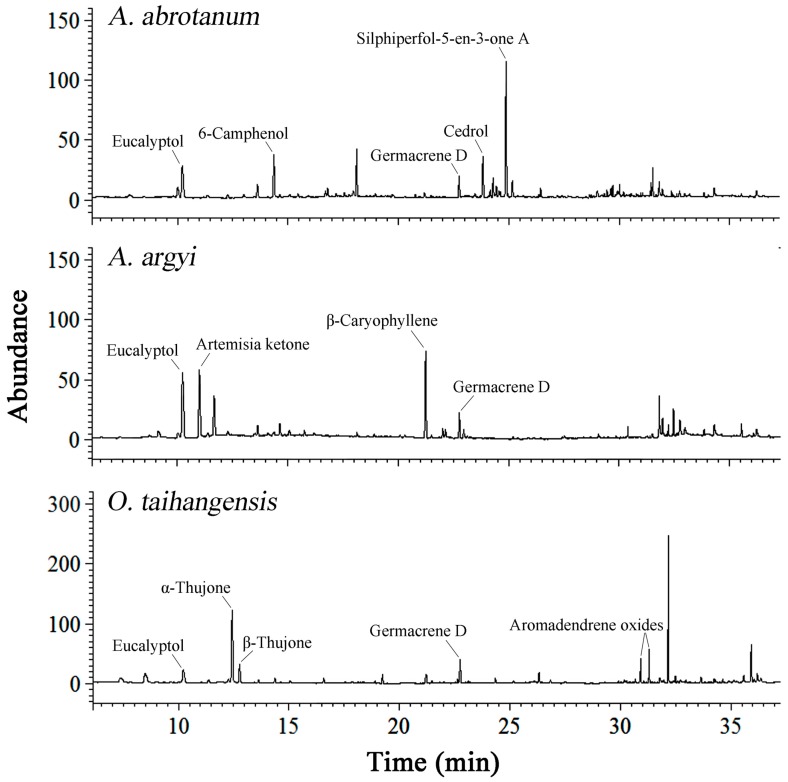
Total ion chromatogram of volatile components in some species of tested plants.

According to GC-MS analysis, 213 constituents were detected and tentatively identified in the n-hexane extracts of 14 samples (Figure 1 and Table S1). The components were classified into nine categories, including four groups of terpenoids (monoterpenes, sesquiterpenes, diterpenes, and oxygenated terpenoids), aromatic compounds, fluorinated compounds, alkanes, alkanols, and others (components such as ketones, enols, and esters, which are not terpenoids). The terpenoids accounted for 117 components and included 12 monoterpenes; 26 sesquiterpenes; one diterpene; and 78 oxygenated terpenoids, consisting of 38 oxygenated monoterpenes or their derivatives, 34 oxygenated sesquiterpenes, four oxygenated diterpenes, and two oxygenated triterpenes. Different quantities of the constituents existed among the tested species (Table 1). The numbers of total components ranged from 11 to 55, with the fewest constituents detected in the leaves of *A. japonica* (**S5**) and *Artemisia sericea* (**S7**) and the most detected in *A. yunnanensis* (**S4**) and *O. taihangensis* (**S14**). The terpenoids in *A. yunnanensis* (**S4**), *O. taihangensis* (**S14**) and *Artemisia abrotanum* (**S6**) were also the most abundant.

**Figure 2:**
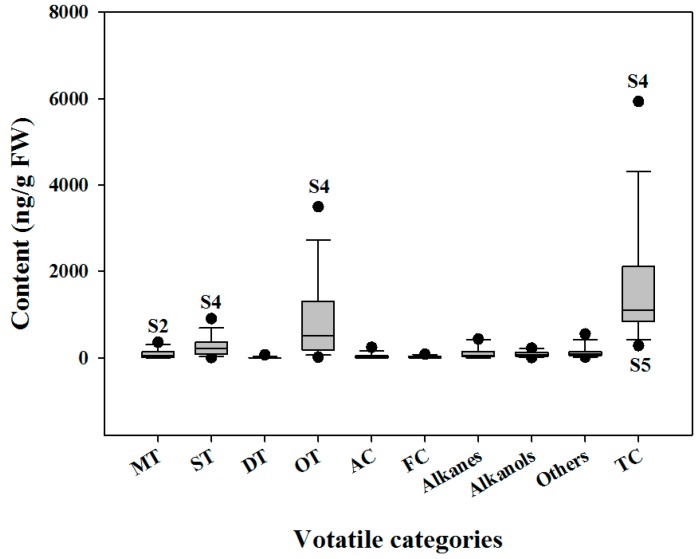
Box plot of the volatile constituents in the leaves of 14 samples. Sample numbers are the same as in Table 2. The abscissa indicates the compound contents of each samples expressed as the mean values of three biologic replicates. The ordinate indicates the volatile categories: MT, monoterpenes; ST, sesquiterpenes; DT, diterpenes; OT, oxygenated terpenes; AC, aromatic compounds; FC, fluorinated compounds; TC, total compounds.

Ethyl decanoate solutions with known concentrations were used for the semi-quantitative analysis of each volatile component and the total compounds (Table S1). The concentrations of oxygenated terpenoids and total compounds varied widely among these species (Figure 2), with a range of 8.20 to 3493.38 ng/g fresh weight (FW) for the former and 275.27 to 5931.41 ng/g FW for the latter. The concentrations of oxygenated terpenoids and total compounds were the lowest in *A. japonica* (**S5**), and its total terpenoid content (170.22 ng/g FW) was also much lower than those of other species. By contrast, *A. yunnanensis* (**S4**) contained the highest levels of sesquiterpenes (900.17 ng/g FW), oxygenated terpenes and total terpenoids. The most abundant monoterpenes (354.10 ng/g FW) were found in the leaves of *A. absinthium* (**S2**). In addition, the leaves of some species such as *A. abrotanum* (**S6**), *A. argyi* (**S8**), *C. indicum* var. *aromaticum* (**S10**), and *O. taihangensis* (**S14**) also contained high total volatile contents that exceeded 1500 ng/g FW. Relatively high levels of total terpenoids, of which concentrations exceeded 1000 ng/g FW, were also detected in these four species. However, the total terpenoid and total compound contents in the leaves of *C. indicum* (**S11**) and *O. longilobus* (**S13**) were much lower than those of their morphologically similar species.

### 2.2. Volatile Composition Patterns in 14 Compositae Plants

**Figure 3:**
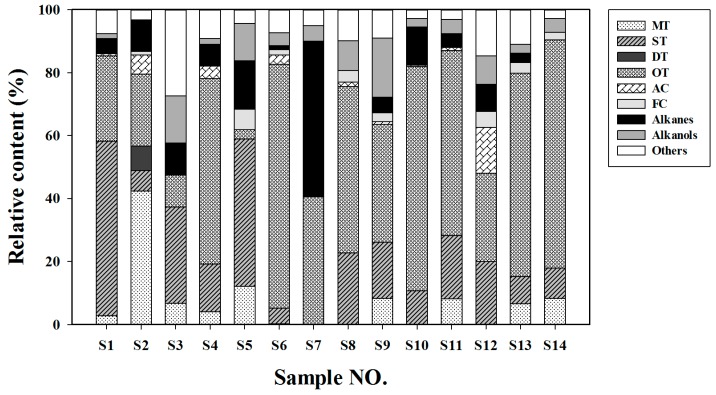
Relative content of volatile constituents in the leaves of 14 samples. Sample numbers are the same as in Table 2. MT, monoterpenes; ST, sesquiterpenes; DT, diterpenes; OT, oxygenated terpenes; AC, aromatic compounds; FC, fluorinated compounds; TC, total compounds.

Although the n-hexane extracts revealed different chemical compositions, the terpenoid components (40.45–90.38%) were generally the predominant compounds in most samples. Figure 3 reveals that except for *A. vulgaris* ‘Variegate’ (**S3**), *A. sericea* (**S7**) and *C. chinense* (**S12**), with lower relative terpenoid contents, the other 11 species contained terpenoid contents that accounted for more than 60% of the total compound contents. Furthermore, oxygenated terpenoids, accounting for 2.98–72.54% of the total compounds, were the main type of terpenoids in most samples. As exceptions, *A. sacrorum* (**S1**), *A. vulgaris* ‘Variegate’ (**S3**) and *A. japonica* (**S5**) contained sesquiterpenes as the predominant terpenoid (55.45%, 30.60%, and 46.68% of the total compounds, respectively), and *A. absinthium* (**S2**) had the highest relative content of monoterpenes (42.29%). Considering the detected oxygenated terpenoids, consisting of oxygenated monoterpenes (0.00% to 70.27% of the total compounds), oxygenated sesquiterpenes (0.00% to 53.53% of the total compounds), oxygenated diterpenes (0.00% to 2.69% of the total compounds) and minor oxygenated triterpenes (0.00% to 3.87% of the total compounds), together with the terpenes, *C. indicum* var. *aromaticum* (**S10**) and *A. yunnanensis* (**S4**) possessed the highest relative contents of monoterpene (70.27%) and sesquiterpene (68.71%) derivatives among the total compounds in their leaves. Diterpene derivatives were only found in the leaves of *A. absinthium* (**S2**), *A. yunnanensis* (**S4**) and *A. abrotanum* (**S6**), accounting for 10.53%, 0.78% and 0.84%, respectively. Negligible amounts of oxygenated triterpenes (0.37%, 3.87%, and 0.48%) were detected in leaves of *A. yunnanensis* (**S4**), *C. chinense* (**S12**), and *O. taihangensis* (**S14**), respectively. The remaining constituents, such as aromatic compounds, also contributing to scent only accounted for 0.00% to 14.53% of the total compounds, with the highest relative content present in the leaves of *C. chinense* (**S12**).

### 2.3. PCA Analysis of 14 Compositae Plants Based on Terpenoid Compounds

**Figure 4:**
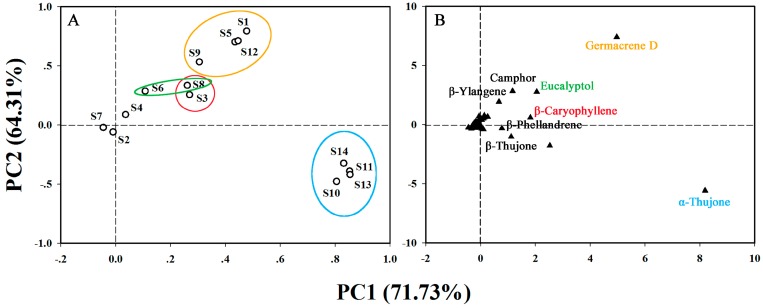
Principal component analysis of 14 samples based on PC1 and PC2 scores. (**A**) Loading plot of samples, and sample numbers are the same as in Table 2; and (**B**) loading plot of terpenoid compounds.

Considering that terpenoid compounds were the predominant components in the tested species, the terpenoid concentrations in the 14 samples were subjected to principal component analysis (PCA) for a better description of these scented plants. Five principal components were obtained, and the accumulated variance of PC1, PC2, PC3, PC4, and PC5 accounted for 71.73%, 64.31%, 56.70%, 46.52%, and 26.23% of the total variance, respectively. Therefore, the first two principal components were chosen to construct a loading diagram. According to the factor scores, the tested samples were positioned in the two-dimensional space, with some obvious groupings (Figure 4A). These groups could be well explained based on the loading spots of the terpenoid components (Figure 4B). The group consisting of *C. indicum* var. *aromaticum* (**S10**), *C. indicum* (**S11**), *O. longilobus* (**S13**), and *O. taihangensis* (**S14**), with the highest PC1 scores and negative PC2 scores, were characterized by high amounts of α-thujone, which accounted for 1432.16 ng/g FW, 552.64 ng/g FW, 283.73 ng/g FW, and 531.48 ng/g FW, respectively. Germacrene D (a sesquiterpene) was the most abundant component of the total terpenoids in the leaves of *A. sacrorum* (**S1**), *A. japonica* (**S5**) and *C. chinense* (**S12**), accounting for 260.91 ng/g FW (31.77%), 77.36 ng/g FW (28.10%), and 80.42 ng/g FW (14.70%), respectively, and grouped these species together with high PC1 and PC2 scores. Close to these three species, *T. vulgare* (**S9**), whose major component was camphor (317.22 ng/g FW, 27.34%), also contained a high percentage of germacrene D (126.68 ng/g FW, 10.92%), followed by β-phellandrene (85.13 ng/g FW, 7.34%), eucalyptol (45.36 ng/g FW, 3.91%) and β-ylangene (40.89 ng/g FW, 3.52%), in its leaves. Another group consisted of *A. vulgaris* ‘Variegate’ (**S3**) and *A. argyi* (**S8**), which were characterized by high concentrations of β-caryophyllene. *A. vulgaris* ‘Variegate’ (**S3**) contained β-caryophyllene (181.14 ng/g FW, 17.35%) as the major terpenoid, followed by β-pinene (62.92 ng/g FW, 6.03%) and *trans*-β-farnesene (59.17 ng/g FW, 5.67%). Similarly, the most abundant terpenoids in the leaves of *A. argyi* (**S8**) included β-caryophyllene (258.69 ng/g FW, 12.40%), eucalyptol (343.57 ng/g FW, 16.47%), and artemisia ketone (293.39 ng/g FW, 14.06%). Eucalyptol (157.46 ng/g FW, 9.93%) was also a main terpenoid constituent in the leaves of *A. abrotanum* (**S6**), which also contained silphiperfol-5-en-3-one A (346.20 ng/g FW, 21.84%), 6-camphenol (140.13 ng/g FW, 8.84%), and cedrol (113.80 ng/g FW, 7.18%) as its main components.

Three samples were located at positions near the original point but had different terpenoid compositions and concentrations (Figure 4A). Among them, *A. absinthium* (**S2**) contained α-phellandrene (175.54 ng/g FW, 20.97%) and β-phellandrene (150.17 ng/g FW, 17.94%) as the predominant compounds. Many terpenoids such as germacrene D, β-phellandrene, arborescin, and β-caryophyllene in the leaves of *A. yunnanensis* (**S4**) were present at high concentrations that exceeded 150 ng/g FW, but the concentration of arglabin (2830.67 ng/g FW, 47.72%) was higher than the concentrations of all other compounds in this sample. The leaf extract of *A. sericea* (**S7**) possessed abundant *trans*-chrysanthenyl acetate (303.35 ng/g FW, 34.87%) and relatively low concentrations of other terpenoid components, such as *cis*-verbenol (31.05 ng/g FW, 3.57%), safranal (13.17 ng/g FW, 1.51%), and tetrahydromyrcenol (4.29 ng/g FW, 0.49%).

### 2.4. HCA Analysis of 14 Compositae Plants Based on Terpenoid Compounds

**Figure 5:**
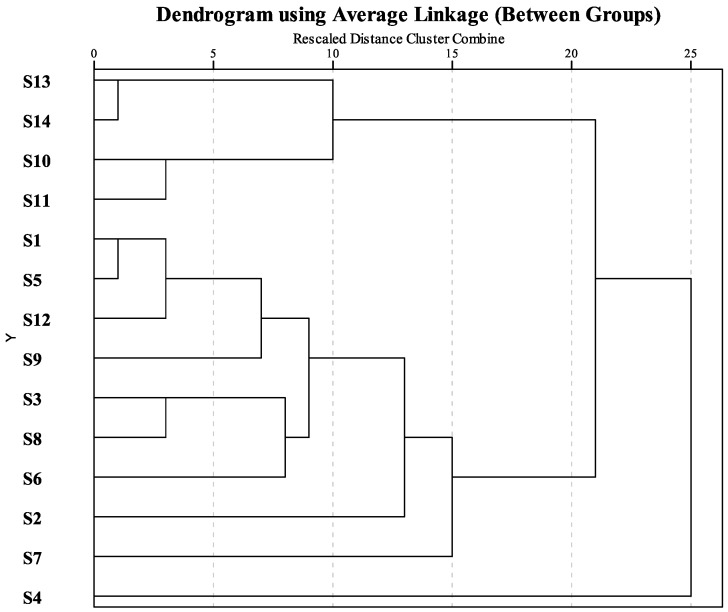
Dendrogram of the hierarchical cluster analysis of 14 samples based on the relative content of terpenoid compounds. The abscissa denotes the Euclidean distances and the ordinate denotes the sample numbers, which are the same as in Table 2.

To classify the tested species by their chemical uniqueness, HCA was performed according to the terpenoid compositions of the 14 samples, the between-group linkage method was used to construct a dendrogram (Figure 5). The species could be classified into several clusters that were similar to the PCA groups. *A. yunnanensis* (**S4**) was isolated because of the extraordinarily high levels of arglabin in its leaves. The four species with high contents of α-thujone were clustered as one group. Interestingly, when the Euclidean distance was set at 10, two sets of varieties, *C. indicum* var. *aromaticum* (**S10**) vs. *C. indicum* (**S11**), and *O. longilobus* (**S13**) vs. *O. taihangensis* (**S14**) clustered together, although the concentrations were extremely different between them. This result demonstrated that these samples had similar chemical compositions.

The remaining plants were initially classified together. However, as the Euclidean distance was decreased, *A. sericea* (**S7**) and *A. absinthium* (**S2**) were gradually separated into single groups. Both of these species contained unique components that were rarely detected in the other samples, e.g., α-phellandrene (20.97%), famesol isomer A (12.57%), geranyl-α-terpinene (7.84%), linalool (3.85%), cis-β-farnesene (3.31%), β-curcumene (3.23%), norethynodrel (2.69%), neryl(S)-2-methylbutanoate (1.76%), and 8-cedren-13-ol (1.49%) in the leaves of *A. absinthium* (**S2**) and the number of terpenoids in *A. sericea* (**S7**). The other linkages, such as the group containing *A. abrotanum* (**S6**), *A. argyi* (**S8**) and *A. vulgaris* ‘Variegate’ (**S3**), and the cluster of *T. vulgare* (**S9**), *A. sacrorum* (**S1**), *A. japonica* (**S5**), and *C. chinense* (**S12**), corresponded well with the PCA loading diagram.

## 3. Discussion

**Figure 6:**
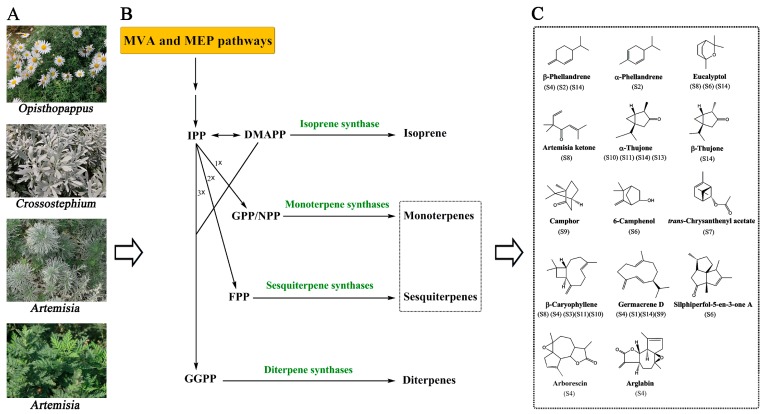
Local diagram of the terpenoid biosynthetic parthway and the chemical structures of the main compounds found in 14 Compositae plants. Graph (**A**) displays the representative samples used in this research; Graph (**B**) was modified according to the literature [37]; and (**C**) the sample numbers which are the same as in Table 2, denote the samples with the highest concentrations of constituents (above 100 ng/g FW).

The chemical constituents, mainly consisting of volatile compounds, in the leaves of 14 Compositae plants with unique scents were investigated qualitatively and quantitatively. Most of the sampled plants contained abundant terpenoids, primarily monoterpenoids, sesquiterpenoids, and derivatives (Figure 6). The terpenoid contents were highest in the leaves of *A. yunnanensis*, *A. abrotanum*, *A. argyi*, *C. indicum* var. *aromaticum*, and *O. taihangensis*. Since monoterpenoids and sesquiterpenoids with lower molecular weight volatilize easily and have specific odors [8,31], these samples have greater potential to be used for extraction of perfume oils. Considering those characteristic constituents (Figure 6C), the tested samples could be defined by their distinct scent via sensory evaluation in a later study, just as *Chrysanthemum* essential oils were assessed based on six sensory attributes including floral, woody, grassy, fruity, sour, and minty [31]. On the other hand, because terpenoids play important roles in protecting plants against pests, these compounds can be divided into different types based on their functions, such as in indirect defense or direct repulsion of pests [32]. For example, eucalyptol and camphor have activity as insect repellents or pesticidal agents [33,34], whereas (*E*)-β-caryophyllene and (*E*)-β-farnesene can additionally attract the natural enemies of herbivores [35,36]. Therefore, the above results could serve as a reference for the utilization of the volatile compounds from these Compositae plants to exploit the specific activities of certain constituents.

In plants, the metabolism of terpenoids use five-carbon isopentenyl diphosphate (IPP) and dimethylallyl pyrophosphate (DMAPP) as precursors, which originate from the mevalonic acid (MVA) and methylerythritol phosphate (MEP) pathways (Figure 6B). One, two, or three units of IPP combine with one unit of DMAPP to generate GPP (or NPP), FPP, and GGPP, respectively, which are subsequently converted to monoterpenes, sesquiterpenes, and diterpenes by the activity of the terpene synthases (TPSs) [37]. Generally, FPP (the precursor of sesquiterpenes) is generated via the MVA pathway in the cytoplasm, whereas GPP and GGPP (the precursor of monoterpenes and diterpenes, respectively) are synthesized from the MEP pathway in plastids [38]. After synthetic processing by TPS enzymes, these terpenes can be further modified by oxidation, hydroxylation, methylation, acylation, or cleavage to form various terpene derivatives [37,38]. For instance, more oxygenated terpenoids than terpenes were detected in many species in our research (Figure 3). Therefore, the diversity of terpenoids in different plant species is due to the action of TPSs and multiple modification of terpenes. The family size and functions of TPSs, which include seven subfamilies are related to species phylogeny [37], suggesting that a diversity of TPSs may exist in different genera of Compositae plants. According to the present research, based on the terpenoid composition in leaves, the genera *Chrysanthemum* and *Opisthopappus* have greater chemical similarity, whereas the *Artemisia* plants, *C. chinense* and *T. vulgare* were classified together. The phylogeny based on the sequence variation in both the nuclear ribosomal (ITS) and chloroplast (trnL-F IGS) DNA constructed by Zhao et al. [39] showed that except for *Tanacetum* (subtribe Tanacetinae), both *Chrysanthemum* and *Opisthopappus* belong to the *Chrysanthemum* group, subtribe Artemisiinae, whereas *Artemisia* and *Crossostephium* fall under the Artemisia group, another part of the subtribe Artemisiinae. Combining the genetic relationships and chemotaxonomy of these plants, insights on the diverse flux and regulation of terpenoid metabolism in different genera of Compositae plants were provided as a foundation for further research.

## 4. Materials and Methods

### 4.1. Plant Materials

The plant materials used in this study included eight *Artemisia* plants, *T. vulgare*, two wild species of *Chrysanthemum* (*C. indicum* and its varieties, *C. indicum* var. *aromaticum*, both of which were originally collected from Shennongjia, Hubei province, China), *C. chinense*, and two species of *Opisthopappus* (Table 2). All plants were cultivated using the same cultivation measures and environmental conditions for several years in the Chrysanthemum Germplasm Resource, Preservation Center, Nanjing Agricultural University, China. Mature leaves were collected in October 2016, and more than three individuals of each species were used as replicates.

### 4.2. Sample Preparation

Fresh leaves of each species were cut into small pieces and accurately weighed. The samples were extracted with HPLC-grade n-hexane (SaFo Technology, Tianjin, China) in a sealed container for 24 h at room temperature. During the extraction process, the samples were shaken several times to increase the extraction efficiency. Next, known concentrations of ethyl decanoate (CAS 110-38-3, ≥98%, Sigma Aldrich, St Louis, MO, USA), an internal standard, were added to portions of the extracts, which were then filtered through 0.22 µm nylon filters prior GC-MS analysis. The experiment was conducted in triplicate.

### 4.3. GC-MS Conditions

The analyses were performed using a GC-MS system (7890A-5975C, Agilent Technologies Inc., Santa Rosa, CA, USA) equipped with an HP-5 MS capillary column (30 m × 0.25 mm, 0.25 mm, Agilent Technologies Inc., Santa Rosa, CA, USA). The injection volume of each sample was 1 μL. Helium (99.999%) was used as the carrier gas at a flow-rate of 1 mL/min. The temperature of the injection port was 250 °C, and the column temperature program was as follows: 50 °C for 2 min, followed by an increase to 180 °C at a rate of 5 °C/min, an increase to 270 °C at a rate of 20 °C/min, and maintenance at 270°C for 5 min. The MS conditions included an EI ion source temperature of 230 °C, an ionization energy of 70 eV, and a mass scan range of 40–500 amu.

### 4.4. Peak Identification

The separated constituents were tentatively identified by comparing their mass spectra with those in the NIST08 MS library (National Institute of Standards and Technology, Gaithersburg, MD, USA) and by comparing their retention indices (RIs) with literature values [2,8,40]. The RIs were calculated relative to a C7-C30 alkane standard (Sigma Aldrich, St Louis, MO, USA) separated on the HP-5 MS capillary column under the same GC-MS analysis conditions [41]. Each constituent was quantified based on the comparison of its peak area with that of the internal standard, and the contents are expressed as the ng/g FW.

### 4.5. Statistical Analysis

Principal component analysis (PCA) based on the contents of the terpenoid constituents was performed to classify the tested species. HCA based on the squared Euclidean distance and the method of between-group linkages were used to cluster the samples with different relative terpenoid contents. Both statistical approaches were conducted using IBM SPSS Statistics 19.0 (IBM, Armonk, NY, USA).

## 5. Conclusions

In this work, the volatile compositions of 14 Compositae plants were measured. Terpenoids, particularly monoterpenoids, sesquiterpenoids, and derivatives, were the most abundant compounds in the extracts of leaves. With a horizontal comparison of the constituents among species or varieties, some samples were characterized by high concentrations of specific compounds that can be further exploited and utilized in future applications. In addition, the phytochemistry analysis in this research lays a foundation for the study of the chemotaxonomy and differential metabolism of species in Compositae.

## Figures and Tables

**Table 1:** Number of volatile categories from leaves of 14 samples.

Categories ^a^	Number of Volatile Categories													
	S1 ^b^	S2	S3	S4	S5	S6	S7	S8	S9	S10	S11	S12	S13	S14
MT	2	3	2	3	2	1	0	1	2	0	4	0	1	3
ST	9	2	4	13	4	3	0	7	4	5	8	3	2	8
DT	0	1	0	0	0	0	0	0	0	0	0	0	0	0
OT	6	7	3	17	1	20	4	12	7	3	7	5	6	20
AC	1	3	0	3	0	1	0	1	2	1	1	3	0	1
FC	0	1	0	0	1	1	0	2	1	0	0	2	1	1
Alkanes	4	2	3	3	1	2	1	0	5	1	2	1	1	0
Alkanols	2	0	3	4	1	2	2	4	2	2	2	2	1	2
Others	2	2	6	12	1	7	4	8	11	4	4	6	5	5
TC	26	21	21	55	11	37	11	35	34	16	28	22	17	40

^a^ MT, monoterpenes; ST, sesquiterpenes; DT, diterpenes; OT, oxygenated terpenes; AC, aromatic compounds; FC, fluorinated compounds; TC, total compounds; ^b^ Sample numbers are the same as in Table 2.

**Table 2:** Compositae plants used in this study.

Sample Number	Accessions	Collection Locality
**S1**	*A. sacrorum*	Dalian, Liaoning province, China
**S2**	*A. absinthium*	Chiba-shi, Japan
**S3**	*A. vulgaris* ‘Variegate’	Nanjing, Jiangsu province, China
**S4**	*A. yunnanensis*	Yunnan province, China
**S5**	*A. japonica*	Hiroshima, Japan
**S6**	*A. abrotanum*	Chiba-shi, Japan
**S7**	*A. sericea*	Nanjing, Jiangsu province, China
**S8**	*A. argyi*	Jiangshan, Zhejiang province, China
**S9**	*T. vulgare*	Tsukuba, Japan
**S10**	*C. indicum* var. *aromaticum*	Shennongjia, Hubei province, China
**S11**	*C. indicum*	Shennongjia, Hubei province, China
**S12**	*C. chinense*	Xiamen, Fujian province, China
**S13**	*O. longilobus*	Yuntai Mountain, Henan province, China
**S14**	*O. taihangensis*	Yuntai Mountain, Henan province, China

## Supplementary Materials

The following are available online, Table S1. Contents of volatile compounds in leaves of 14 Compositae plants.
