# Supplementary material for: GC-MS Analysis of the Volatile Constituents in the Leaves of 14 Compositae Plants
Source: Molecules. 2018 Jan 18;23(1):166. doi: 10.3390/molecules23010166 (PMC6016956; doi:10.3390/molecules23010166)
Supplement: Supplementary file 1 [file molecules-23-00166-s001.pdf]

Table S1. Contents of volatile compounds in leaves of 14 Compositae plants.

RI	Compounds	Type
902	Santolina triene	MT
932	α-Pinene	MT
948	Camphene	MT
971	β-Phellandrene	MT
976	β-Pinene	MT
988	(1S)-(-)-β-Pinene	MT
1005	α-Phellandrene	MT
1035	*trans* -β-Ocimene	MT
1047	Ocimene	MT
1058	γ-Terpinene	MT
1085	4-Carene	MT
1128	Alloocimene	MT
1333	Elixene	ST
1377	α-Copaene	ST
1390	Berkheyaradulene	ST
1420	β-Ylangene	ST
1422	β-Caryophyllene	ST
1432	Cubebene	ST
1437	β-Copaene A	ST
1449	ε-Muurolene	ST
1451	γ-Cadinene	ST
1452	*trans* -β-Farnesene	ST
1457	α-Caryophyllene	ST
1457	1, 4, 7-Cyclopentadiene, 1, 5, 9, 9-tetramethyl-, z, z, z	ST
1467	α-Cubebene	ST
1474	β-curcumene	ST
1478	Himachalene	ST
1478	*cis* -β-Farnesene	ST
1482	Germacrene D	ST
1482	β-Copaene B	ST
1490	β-Eudesmene	ST
1492	Germacrene B	ST
1494	Zingiberene	ST
1497	γ-Elemene	ST
1497	a-Ylangene	ST
1503	α-Farnesene	ST
1524	β-Sesquiphellandrene	ST
1541	α-Caryophyllene	ST
1988	Geranyl-α-terpinene	DT
978	Linalool propionate	OT
1031	Eucalyptol	OT
1057	Artemisia ketone	OT
1068	Lilac alcohol	OT
1069	*cis* -β-Terpineol	OT
1079	1,5-Heptadien-4-ol,3,3,6-trimethyl-	OT
1096	Linalool	OT
1099	*cis* -p-Menth-2-en-1-ol	OT
1105	α-Thujone	OT
1117	β-Thujone	OT
1138	Verbenol	OT
1146	Camphor	OT
1154	Umbellulon	OT

1161	*cis* -Verbenol	OT
1169	(-)-α-Terpineol	OT
1170	6-Camphenol	OT
1171	Borneol	OT
1180	4-Terpineol	OT
1195	α-Terpineol	OT
1202	Verbenone	OT
1203	*cis* -Sabinol	OT
1209	*trans* -Piperitol	OT
1219	*cis* -Carveol	OT
1240	Sabinyl acetate	OT
1243	Carvone	OT
1248	Linalyl formate	OT
1252	Piperitone epoxide	OT
1256	7-Oxabicyclo[4.1.0]heptane-2-one,3-methyl-6-(1-methylethyl)-	OT
1256	*trans* -Chrysanthenyl acetate	OT
1283	L-Borneol acetate	OT
1290	1,4-dihydroxy-p-menth-2-ene	OT
1346	Terpinyl acetate	OT
1350	4-Pinanol (6CI,7CI,8CI)	OT
1417	2-Cyclohexen-1-one,4-hydroxy-3-methyl-6-(1-methylethyl)- A	OT
1425	2-Cyclohexen-1-one,4-hydroxy-3-methyl-6-(1-methylethyl)- B	OT
1526	Cedrol	OT
1538	(3R)-2,2,5aβ,9β-Tetramethyl-3β,9aβ-methanodecahydro-1-benzoxepin	OT
1545	Silphiperfol-5-en-3-one B	OT
1548	α-Elemol	OT
1548	Hedycaryol	OT
1551	Silphiperfol-5-en-3-ol	OT
1556	5.alpha-Hydroxy-4.alpha,8,10,11-tetramethyltricyclo[6,3,0,0(2,40]	OT
1565	Neryl(S)-2-methylbutanoate	OT
1570	Silphiperfol-5-en-3-one A	OT
1577	Germacrene D-4-ol	OT
1582	Longiverbenone	OT
1583	Caryophyllene oxide	OT
1618	Safranal	OT
1632	(+)-γ-Eudesmol	OT
1635	Humulane-1,6-dien-3-ol	OT
1637	Tetracyclo[6.3.2.0(2,5).0(1,8)]tridecan-9-ol,4,4-dimethyl-	OT
1655	β-Eudesmol	OT
1685	trans-Sesquisabinene hydrate	OT
1685	α-Bisabolol	OT
1772	6-(1-Hydroxymethylvinyl)-4,8a-dimethyl-3,5,6,7,8,8a-hexahydro-1H-naphthale	OT
1844	Retinal	OT
1872	α-Damascone	OT
1776	8-Cedren-13-ol	OT
1928	Pentandioic acid,(p-t-butylphenyl)ester	OT
1958	Aromadendrene oxide A	OT
1975	Bisabolol oxide A	OT
1982	Arborescin	OT
2001	8-Cedren-13-ol	OT
2009	Aromadendrene oxide B	OT
2019	trans-6-(p-Tolyl)-2-methyl-2-heptenol	OT
2027	Z-2-(9-Octadecenyloxy)-ethanol	OT
2043	Arglabin	OT
2071	Norethynodrel	OT
2083	2,2,7,7-Tetramethyltricyclo[6.2.10(1,6)]undec-4-en-3-one	OT
2113	cis-Lanceol	OT

2127	Tricyclo[20.8.0.0(7,16)]triacontane,1(22),7(16)-diepoxy-	OT
2150	2-Furfurylidene-3,4,4a,5,6,7,8,8aβ-octahydronaphthalen-1(2H)-one	OT
2189	Confertin	OT
2202	Deoxynivalenol (Vomitoxin)	OT
2220	Famesol isomer A	OT
2225	Tetrahydromyrcenol	OT
2331	Chrysanin	OT
2361	Germanicol	OT
1024	o-Cymene	AC
1099	Hydrocinnamic acid	AC
1225	p-Cumenol	AC
1515	2-Methoxyhydroquinone	AC
1527	4-Methoxycatechol	AC
1572	3-Methoxythiophenol	AC
1792	3,4,5,6-Tetrachlorophthalonitrile	AC
1846	Diisobutyl phthalate	AC
1892	Isophthalic acid, ethyl tridec-2-ynyl ester	AC
1944	Phthalic acid, 8-chlorooctyl isobutyl ester	AC
1957	Geranyl-p-cymene	AC
2037	2(1H)-Naphthalenone, octahydro-4a-phenyl-, trans	AC
2254	Hexatriacontyl pentafluoropropionate	FC
2247	Octariacontyl pentafluoropropionate	FC
2495	Octaconyl trifluoroacetate	FC
2496	Docosyl pentafluoropropionate	FC
2699	Tetratriacontyl pentafluoropropionate	FC
2700	Tetratriacontyl heptafluorobutyrate	FC
1343	1-Nonylcycloheptane	Alkanes
1700	Heptadecane	Alkanes
1900	Nonadecane	Alkanes
1913	Tetradecyloxirane	Alkanes
2000	Eicosane	Alkanes
2225	2-Hexadecyloxirane	Alkanes
2307	1-Chlorooctadecane	Alkanes
2443	2-methyltetracosane	Alkanes
2800	Octacosane	Alkanes
3100	Hentriacontane	Alkanes
4300	Tritetracontane	Alkanes
4391	3,5,24-Trimethyltetracontane	Alkanes
996	3-Octanol	Alkanols
2088	1-Nonadecanol	Alkanols
2088	1-Heptadecanol	Alkanols
2090	1-Hexadecanol	Alkanols
2196	1-Heptatriacotanol	Alkanols
2293	2-Hexyl-1-decanol	Alkanols
2494	Behenyl alcohol	Alkanols
2495	1-Heptacosanol	Alkanols
2497	1-Heneicosanol	Alkanols
2606	1-Octacosanol	Alkanols
978	1-Hepten-3-ol	Others
979	1-Octen-3-ol	Others
1179	3-Cyclohexene-1-methanol	Others
1234	Sulfurous acid, 2-ethylhexyl tridecyl ester	Others
1234	3-Methyl-2-butenoic acid,oct-3-en-2-yl ester	Others
1271	*cis* -2-Methyl-4-n-pentylthiane,S,S-dioxide	Others
1314	1,3-Cyclopentadiene,5,5-dimethyl-1-ethyl	Others
1324	*trans* -2-Methyl-4-n-pentylthiane,S,S-dioxide	Others
1327	Phytene	Others

1364	Tricyclo[6.3.0.0(2.7)]undecane-1,3,7-triol	Others
1453	5,9-Undecadien-1-yne,6,10-dimethyl-	Others
1492	Eicosanoic acid, ethylester	Others
1493	Undecanoic acid, ethyl ester	Others
1493	Palmitic acid ethyl ester	Others
1499	2,4-Di-tert-butylphenol	Others
1573	Butyl6,9,12,15-octadecatetraenoate	Others
1636	Adipic acid,2-decyl isobutyl ester	Others
1640	Methyl jasmonate	Others
1672	Cyclopentaneacetic acid,3-oxo-2-(2-pentenyl)-,methyl ester	Others
1677	Tetradecanal	Others
1749	a-Methoxybenzeneaceticacid	Others
1800	17-epi-Methandrostenolone	Others
1824	(Z)-7-Hexadecenal	Others
1839	Acetic acid,[4-(1-hydroxy-1-methylethyl)cyclohex-1-enyl]methyl	Others
1847	(E)-3-Eicosene	Others
1890	cis,cis-7,10-Hexadecadienal	Others
1894	7,9-Di-tert-butyl-1-oxaspiro(4,5)deca-6,9-diene-2,8-dione	Others
1894	2-hydroxy-9,12,15-Octadecatrienoic acid, methyl ester	Others
1895	Linolenic acid	Others
1921	Methyl 14-methylpentadecanoate	Others
1952	(E,E,E)-3,7,11,15-Tetramethylhexadeca-1,3,6,10,14-pentaene	Others
1962	Cycloisolongifolene, 8, 9-dehydro-9-formyl-	Others
1972	*cis* -13-Eicosenoic acid	Others
2007	Butyl-9,12,15-octadecatrienoate	Others
2013	1-Pentadecanal	Others
2019	(Z)-2-Octadecen-1-ol	Others
2089	Oleyl chloride	Others
2106	2-(7-Heptadecynyloxy)tetrahydro-2H-pyran	Others
2111	Phytol	Others
2126	13-Methyltetradec-9-enoic acid methyl ester	Others
2161	16-methyloxacyclohexadeca-3,5-dien-2-one	Others
2224	1-Androstenedione isomer	Others
2224	α-L-Rhamnose	Others
2227	2-Pentacosanone	Others
2249	Sulfurous acid,butyl heptadecyl ester	Others
2291	1-Hexacosene	Others
2292	1-Docosene	Others
2292	Isobutyl tetradecyl carbonate	Others
2319	Hexacosyl acetate	Others
2380	Fumaric acid, heptadecyl trans-hex-3-enyl ester	Others
2429	1,2-Epoxynonadecane	Others
2634	(Z)-2-Octadecen-1-ol	Others
2670	Cyclobutanecarboxylic acid,tridec-2-ynyl ester	Others
2672	(Z)-2-(9-Octadecenyloxy)-ethanol	Others
2683	3-Methyl-2-butenoic acid, tridec-2-ynyl ester	Others
2786	Cycloartenyl acetate	Others
	Total compounds	

[a] The content of each compound was the mean of three replicate analysis.

[b] Sample numbers are the same as in Table 2.

Chemical formula	Content (ng/g, FW) [a]									
	S1 [b]	S2	S3	S4	S5	S6	S7	S8	S9	S10
C10H16	0.00	0.00	0.00	3.05	0.00	0.00	0.00	0.00	0.00	0.00
C10H16	0.00	0.00	7.15	0.00	0.00	0.00	0.00	1.52	0.00	0.00
C10H16	0.00	0.00	0.00	0.00	0.00	3.31	0.00	0.00	11.39	0.00
C10H16	0.00	150.17	0.00	212.12	0.00	0.00	0.00	0.00	85.13	0.00
C10H16	0.00	0.00	62.92	0.00	0.00	0.00	0.00	0.00	0.00	0.00
C10H16	5.03	28.38	0.00	0.00	13.21	0.00	0.00	0.00	0.00	0.00
C10H16	0.00	175.54	0.00	25.17	0.00	0.00	0.00	0.00	0.00	0.00
C10H16	0.00	0.00	0.00	0.00	0.00	0.00	0.00	0.00	0.00	0.00
C10H16	17.73	0.00	0.00	0.00	20.31	0.00	0.00	0.00	0.00	0.00
C10H16	0.00	0.00	0.00	0.00	0.00	0.00	0.00	0.00	0.00	0.00
C10H16	0.00	0.00	0.00	0.00	0.00	0.00	0.00	0.00	0.00	0.00
C10H16	0.00	0.00	0.00	0.00	0.00	0.00	0.00	0.00	0.00	0.00
C15H24	17.16	0.00	0.00	23.04	0.00	0.00	0.00	0.00	14.26	0.00
C15H24	17.71	0.00	0.00	20.95	0.00	0.00	0.00	0.00	0.00	0.00
C15H24	0.00	0.00	0.00	24.27	0.00	0.00	0.00	0.00	0.00	0.00
C15H24	72.39	0.00	0.00	0.00	19.62	12.81	0.00	0.00	40.89	0.00
C15H24	0.00	0.00	181.14	182.76	0.00	0.00	0.00	258.69	0.00	115.70
C15H24	26.58	0.00	0.00	43.99	9.66	6.70	0.00	33.68	0.00	15.36
C15H24	13.53	0.00	0.00	0.00	0.00	0.00	0.00	0.00	25.03	0.00
C15H24	16.68	0.00	0.00	0.00	0.00	0.00	0.00	0.00	0.00	0.00
C15H24	0.00	0.00	0.00	19.93	0.00	0.00	0.00	0.00	0.00	0.00
C15H24	0.00	0.00	59.17	0.00	0.00	0.00	0.00	30.81	0.00	0.00
C15H24	0.00	0.00	33.33	0.00	0.00	0.00	0.00	38.16	0.00	12.70
C15H24	0.00	0.00	0.00	103.73	0.00	0.00	0.00	0.00	0.00	0.00
C15H24	0.00	0.00	0.00	0.00	0.00	0.00	0.00	0.00	0.00	0.00
C15H24	0.00	27.06	0.00	0.00	0.00	0.00	0.00	0.00	0.00	0.00
C15H24	0.00	0.00	0.00	0.00	0.00	0.00	0.00	0.00	0.00	0.00
C15H24	0.00	27.69	0.00	0.00	0.00	0.00	0.00	0.00	0.00	0.00
C15H24	260.91	0.00	45.81	283.24	77.36	59.42	0.00	70.42	126.68	81.01
C15H24	14.18	0.00	0.00	0.00	0.00	0.00	0.00	0.00	0.00	0.00
C15H24	0.00	0.00	0.00	98.20	0.00	0.00	0.00	28.97	0.00	0.00
C15H24	16.22	0.00	0.00	0.00	0.00	0.00	0.00	0.00	0.00	0.00
C15H24	0.00	0.00	0.00	12.89	0.00	0.00	0.00	0.00	0.00	0.00
C15H24	0.00	0.00	0.00	17.80	0.00	0.00	0.00	0.00	0.00	0.00
C15H24	0.00	0.00	0.00	15.25	0.00	0.00	0.00	12.77	0.00	0.00
C15H24	0.00	0.00	0.00	0.00	21.87	0.00	0.00	0.00	0.00	7.43
C15H24	0.00	0.00	0.00	54.13	0.00	0.00	0.00	0.00	0.00	0.00
C15H24	0.00	0.00	0.00	0.00	0.00	0.00	0.00	0.00	0.00	0.00
C20H32	0.00	65.67	0.00	0.00	0.00	0.00	0.00	0.00	0.00	0.00
C13H22O2	0.00	0.00	0.00	42.91	0.00	0.00	0.00	0.00	0.00	0.00
C10H13O	30.09	0.00	38.35	14.55	0.00	157.46	0.00	343.57	45.36	0.00
C10H16O	0.00	0.00	0.00	0.00	0.00	0.00	0.00	293.39	0.00	0.00
C10H18O2	0.00	0.00	0.00	0.00	0.00	0.00	0.00	0.00	0.00	0.00
C10H18O	0.00	0.00	0.00	0.00	0.00	0.00	0.00	22.43	0.00	0.00
C10H18O	0.00	0.00	0.00	0.00	0.00	0.00	0.00	170.54	0.00	0.00
C10H18O	0.00	32.23	0.00	0.00	0.00	0.00	0.00	0.00	0.00	0.00
C10H18O	0.00	0.00	0.00	0.00	0.00	16.98	0.00	0.00	0.00	0.00
C10H16O	0.00	1.81	0.00	0.00	0.00	0.00	0.00	0.00	0.00	1432.16
C10H16O	0.00	2.13	0.00	0.00	0.00	0.00	0.00	0.00	0.00	90.67
C10H16O	23.65	0.00	0.00	0.00	0.00	0.00	0.00	0.00	0.00	0.00
C10H16O	97.54	0.00	0.00	0.00	0.00	51.38	0.00	50.25	317.22	0.00
C10H14O	0.00	0.00	0.00	0.00	0.00	0.00	0.00	0.00	0.00	0.00

C10H16O	21.70	0.00	0.00	0.00	0.00	0.00	31.05	0.00	17.86	0.00
C10H18O	0.00	0.00	0.00	0.00	0.00	0.00	0.00	14.62	0.00	0.00
C10H16O	0.00	0.00	0.00	0.00	0.00	140.13	0.00	0.00	16.47	0.00
C10H18O	0.00	0.00	0.00	0.00	0.00	0.00	0.00	19.98	15.40	0.00
C10H18O	0.00	0.00	0.00	0.00	0.00	13.11	0.00	51.03	0.00	0.00
C10H18O	0.00	0.00	0.00	21.30	0.00	0.00	0.00	35.81	0.00	0.00
C10H14O	29.48	0.00	0.00	0.00	0.00	0.00	0.00	0.00	0.00	0.00
C10H16O	0.00	0.00	0.00	24.97	0.00	0.00	0.00	0.00	0.00	0.00
C10H18O	0.00	0.00	0.00	0.00	0.00	16.36	0.00	0.00	0.00	0.00
C10H16O	0.00	0.00	0.00	0.00	0.00	0.00	0.00	28.96	0.00	0.00
C12H18O2	0.00	0.00	0.00	16.58	0.00	0.00	0.00	0.00	0.00	0.00
C10H14O	0.00	0.00	0.00	0.00	0.00	0.00	0.00	0.00	4.41	0.00
C11H18O2	0.00	0.00	0.00	0.00	0.00	0.00	0.00	0.00	0.00	0.00
C10H16O2	0.00	0.00	0.00	0.00	0.00	21.24	0.00	0.00	0.00	0.00
C10H16O2	0.00	0.00	0.00	0.00	0.00	28.51	0.00	0.00	0.00	0.00
C12H18O2	0.00	0.00	0.00	0.00	0.00	0.00	303.35	0.00	0.00	0.00
C12H20O2	0.00	0.00	0.00	0.00	0.00	15.97	0.00	0.00	0.00	0.00
C10H18O2	0.00	0.00	0.00	0.00	0.00	10.69	0.00	0.00	0.00	0.00
C12H20O2	0.00	0.00	0.00	0.00	0.00	0.00	0.00	0.00	0.00	0.00
C10H18O	0.00	0.00	0.00	86.43	0.00	0.00	0.00	0.00	0.00	0.00
C10H16O2	0.00	0.00	0.00	25.37	0.00	0.00	0.00	0.00	0.00	0.00
C10H16O2	0.00	0.00	0.00	18.20	0.00	0.00	0.00	0.00	0.00	0.00
C15H26O	0.00	0.00	0.00	0.00	0.00	113.80	0.00	0.00	0.00	0.00
C15H26O	0.00	0.00	0.00	0.00	0.00	0.00	0.00	0.00	0.00	19.84
C15H22O	0.00	0.00	0.00	0.00	0.00	55.19	0.00	0.00	0.00	0.00
C15H26O	0.00	0.00	0.00	0.00	0.00	0.00	0.00	0.00	0.00	0.00
C15H26O	0.00	0.00	0.00	0.00	0.00	0.00	0.00	0.00	0.00	0.00
C15H23O	0.00	0.00	0.00	0.00	0.00	37.26	0.00	0.00	0.00	0.00
C15H24O	0.00	0.00	0.00	0.00	0.00	41.73	0.00	0.00	0.00	0.00
C15H26O2	0.00	14.76	0.00	0.00	0.00	0.00	0.00	0.00	0.00	0.00
C15H22O	0.00	0.00	0.00	0.00	0.00	346.20	0.00	0.00	0.00	0.00
C15H26O	20.09	0.00	26.37	29.99	8.20	0.00	0.00	0.00	0.00	0.00
C15H22O	0.00	0.00	0.00	0.00	0.00	57.67	0.00	0.00	0.00	0.00
C15H24O	0.00	0.00	0.00	39.47	0.00	0.00	0.00	0.00	0.00	0.00
C10H14O	0.00	0.00	0.00	0.00	0.00	0.00	13.17	0.00	0.00	0.00
C15H26O	0.00	0.00	0.00	0.00	0.00	0.00	0.00	0.00	0.00	0.00
C15H26O	0.00	0.00	40.75	0.00	0.00	0.00	0.00	0.00	0.00	0.00
C15H24O	0.00	0.00	0.00	0.00	0.00	47.63	0.00	0.00	0.00	0.00
C15H26O	0.00	0.00	0.00	0.00	0.00	0.00	0.00	0.00	0.00	0.00
C15H26O	0.00	0.00	0.00	30.27	0.00	0.00	0.00	0.00	0.00	0.00
C15H26O	0.00	0.00	0.00	0.00	0.00	0.00	0.00	0.00	18.04	0.00
C15H22O2	0.00	0.00	0.00	0.00	0.00	31.47	0.00	0.00	0.00	0.00
C20H28O	0.00	0.00	0.00	0.00	0.00	13.31	0.00	0.00	0.00	0.00
C13H20O	0.00	0.00	0.00	0.00	0.00	0.00	0.00	0.00	0.00	0.00
C15H24O	0.00	0.00	0.00	0.00	0.00	0.00	0.00	0.00	0.00	0.00
C15H20O4	0.00	0.00	0.00	0.00	0.00	0.00	0.00	0.00	0.00	0.00
C15H24O	0.00	0.00	0.00	0.00	0.00	0.00	0.00	0.00	0.00	0.00
C15H26O2	0.00	0.00	0.00	0.00	0.00	10.81	0.00	0.00	0.00	0.00
C15H20O3	0.00	0.00	0.00	210.69	0.00	0.00	0.00	0.00	0.00	0.00
C15H24O	0.00	12.52	0.00	0.00	0.00	0.00	0.00	0.00	0.00	0.00
C15H24O	0.00	0.00	0.00	0.00	0.00	0.00	0.00	0.00	0.00	0.00
C15H20O	0.00	0.00	0.00	34.04	0.00	0.00	0.00	0.00	0.00	0.00
C20H40O2	0.00	0.00	0.00	23.55	0.00	0.00	0.00	0.00	0.00	0.00
C15H18O3	0.00	0.00	0.00	2830.67	0.00	0.00	0.00	17.52	0.00	0.00
C20H26O2	0.00	22.52	0.00	0.00	0.00	0.00	0.00	0.00	0.00	0.00
C15H22O	0.00	0.00	0.00	0.00	0.00	0.00	0.00	0.00	0.00	0.00
C15H24O	0.00	0.00	0.00	0.00	0.00	0.00	0.00	0.00	0.00	0.00

C30H52O2	0.00	0.00	0.00	21.91	0.00	0.00	0.00	0.00	0.00	0.00
C15H18O2	0.00	0.00	0.00	0.00	0.00	0.00	0.00	0.00	0.00	0.00
C15H20O3	0.00	0.00	0.00	0.00	0.00	0.00	0.00	0.00	0.00	0.00
C15H20O6	0.00	0.00	0.00	0.00	0.00	0.00	0.00	52.34	0.00	0.00
C15H26O	0.00	105.21	0.00	0.00	0.00	0.00	0.00	0.00	0.00	0.00
C10H22O	0.00	0.00	0.00	0.00	0.00	0.00	4.29	0.00	0.00	0.00
C20H26O5	0.00	0.00	0.00	22.49	0.00	0.00	0.00	0.00	0.00	0.00
C30H50O	0.00	0.00	0.00	0.00	0.00	0.00	0.00	0.00	0.00	0.00
C10H14	0.00	18.95	0.00	201.44	0.00	48.50	0.00	30.52	0.00	9.65
C9H10O2	5.39	0.00	0.00	0.00	0.00	0.00	0.00	0.00	0.00	0.00
C9H12O	0.00	0.00	0.00	28.33	0.00	0.00	0.00	0.00	0.00	0.00
C7H8O3	0.00	0.00	0.00	0.00	0.00	0.00	0.00	0.00	0.00	0.00
C7H8O3	0.00	0.00	0.00	0.00	0.00	0.00	0.00	0.00	0.00	0.00
C7H8O3	0.00	0.00	0.00	0.00	0.00	0.00	0.00	0.00	0.00	0.00
C8Cl4N2	0.00	0.00	0.00	0.00	0.00	0.00	0.00	0.00	0.00	0.00
C16H22O4	0.00	0.00	0.00	0.00	0.00	0.00	0.00	0.00	4.37	0.00
C23H32O4	0.00	4.07	0.00	0.00	0.00	0.00	0.00	0.00	0.00	0.00
C20H29ClO4	0.00	0.00	0.00	0.00	0.00	0.00	0.00	0.00	5.46	0.00
C18H26	0.00	0.00	0.00	6.47	0.00	0.00	0.00	0.00	0.00	0.00
C16H20O	0.00	27.51	0.00	0.00	0.00	0.00	0.00	0.00	0.00	0.00
C39H73F5O2	0.00	0.00	0.00	0.00	0.00	0.00	0.00	40.42	0.00	0.00
C41H77F5O2	0.00	0.00	0.00	0.00	0.00	0.00	0.00	0.00	32.89	0.00
C30H57F3O2	0.00	0.00	0.00	0.00	0.00	26.00	0.00	0.00	0.00	0.00
C25H45F5O2	0.00	0.00	0.00	0.00	18.08	0.00	0.00	0.00	0.00	0.00
C37H69F5O2	0.00	9.80	0.00	0.00	0.00	0.00	0.00	0.00	0.00	0.00
C38H69F7O2	0.00	0.00	0.00	0.00	0.00	0.00	0.00	35.15	0.00	0.00
C16H32	0.00	0.00	0.00	69.47	0.00	0.00	0.00	0.00	0.00	0.00
C17H36	1.32	0.00	0.00	0.00	0.00	0.00	0.00	0.00	0.00	0.00
C19H40	0.00	0.00	0.00	0.00	0.00	0.00	0.00	0.00	4.71	0.00
C16H32O	0.00	0.00	0.00	0.00	0.00	0.00	0.00	0.00	5.31	0.00
C20H42	5.66	0.00	0.00	0.00	0.00	0.00	0.00	0.00	0.00	0.00
C18H36O	0.00	0.00	0.00	0.00	0.00	5.97	0.00	0.00	23.55	0.00
C18H37Cl	0.00	0.00	0.00	60.88	0.00	0.00	0.00	0.00	0.00	0.00
C25H52	0.00	0.00	12.68	0.00	0.00	0.00	0.00	0.00	0.00	0.00
C28H58	0.00	74.48	8.37	0.00	0.00	0.00	0.00	0.00	0.00	0.00
C31H64	33.01	0.00	85.59	277.60	42.14	15.00	431.18	0.00	12.34	261.86
C43H88	0.00	9.48	0.00	0.00	0.00	0.00	0.00	0.00	0.00	0.00
C43H88	0.00	0.00	0.00	0.00	0.00	0.00	0.00	0.00	11.56	0.00
C8H18O	0.00	0.00	0.00	16.45	0.00	0.00	0.00	32.64	0.00	0.00
C19H40O	0.00	0.00	0.00	0.00	0.00	34.39	0.00	0.00	0.00	31.77
C17H36O	0.00	0.00	123.20	0.00	32.59	0.00	25.33	90.08	0.00	0.00
C16H34O	7.18	0.00	0.00	0.00	0.00	0.00	0.00	28.21	0.00	0.00
C37H76O	0.00	0.00	0.00	18.63	0.00	0.00	0.00	0.00	0.00	0.00
C16H34O	4.91	0.00	0.00	0.00	0.00	0.00	0.00	0.00	55.30	0.00
C22H46O	0.00	0.00	0.00	0.00	0.00	28.99	0.00	0.00	0.00	0.00
C27H56O	0.00	0.00	0.00	31.37	0.00	0.00	17.65	0.00	0.00	28.46
C21H44O	0.00	0.00	24.54	39.41	0.00	0.00	0.00	48.01	162.47	0.00
C28H58O	0.00	0.00	8.75	0.00	0.00	0.00	0.00	0.00	0.00	0.00
C7H14O	0.00	3.98	0.00	0.00	0.00	0.00	0.00	0.00	0.00	0.00
C8H16O	0.00	0.00	0.00	0.00	0.00	0.00	0.00	2.40	0.00	0.00
C7H12O	0.00	0.00	0.00	0.00	0.00	0.00	0.00	0.00	0.00	0.00
C21H44O3S	1.21	0.00	0.00	0.00	0.00	0.00	0.00	0.00	0.00	0.00
C13H22O2	0.00	0.00	0.00	0.00	0.00	0.00	0.00	18.45	0.00	0.00
C11H22O2S	0.00	0.00	0.00	23.74	0.00	0.00	0.00	0.00	0.00	0.00
C9H14	0.00	0.00	24.87	0.00	0.00	0.00	0.00	0.00	0.00	0.00
C11H22O2S	0.00	0.00	0.00	106.95	0.00	0.00	0.00	0.00	0.00	0.00
C20H40	0.00	0.00	0.00	56.47	0.00	0.00	0.00	0.00	0.00	0.00

C13H22O3	0.00	0.00	0.00	0.00	0.00	18.64	0.00	0.00	0.00	0.00
C13H20	0.00	0.00	0.00	38.37	0.00	0.00	0.00	0.00	0.00	0.00
C22H44O2	0.00	0.00	0.00	0.00	0.00	0.00	0.00	0.00	0.00	0.00
C13H26O2	0.00	0.00	0.00	0.00	12.23	0.00	4.69	0.00	0.00	6.54
C18H36O2	0.00	0.00	0.00	0.00	0.00	0.00	0.00	0.00	8.70	0.00
C14H22O	38.90	0.00	0.00	0.00	0.00	0.00	0.00	0.00	0.00	0.00
C22H36O2	0.00	0.00	0.00	0.00	0.00	15.77	0.00	0.00	0.00	0.00
C20H38O4	0.00	0.00	0.00	0.00	0.00	0.00	0.00	0.00	3.39	0.00
C13H20O3	0.00	0.00	0.00	0.00	0.00	0.00	0.00	0.00	0.00	0.00
C13H20O3	0.00	0.00	0.00	0.00	0.00	0.00	0.00	0.00	0.00	0.00
C14H28O	0.00	0.00	0.00	0.00	0.00	0.00	0.00	0.00	4.22	0.00
C9H10O3	0.00	0.00	0.00	0.00	0.00	13.06	0.00	0.00	0.00	0.00
C19H26O2	0.00	0.00	0.00	0.00	0.00	26.55	0.00	0.00	0.00	0.00
C16H30O	0.00	0.00	0.00	15.83	0.00	0.00	0.00	0.00	0.00	0.00
C12H20O3	0.00	0.00	0.00	0.00	0.00	0.00	0.00	0.00	0.00	0.00
C20H40	0.00	0.00	0.00	0.00	0.00	0.00	0.00	0.00	0.00	0.00
C16H28O	0.00	0.00	0.00	0.00	0.00	0.00	0.00	5.94	0.00	0.00
C17H24O3	0.00	0.00	0.00	0.00	0.00	0.00	0.00	0.00	7.00	0.00
C19H32O3	0.00	0.00	0.00	0.00	0.00	0.00	0.00	0.00	0.00	0.00
C18H30O2	0.00	0.00	0.00	0.00	0.00	0.00	0.00	31.39	0.00	0.00
C17H34O2	0.00	0.00	0.00	0.00	0.00	0.00	0.00	0.00	3.20	0.00
C20H32	0.00	23.31	0.00	0.00	0.00	0.00	0.00	0.00	0.00	0.00
C16H22O	0.00	0.00	0.00	21.17	0.00	0.00	0.00	0.00	0.00	0.00
C20H38O2	0.00	0.00	0.00	0.00	0.00	0.00	0.00	0.00	6.30	0.00
C22H38O2	0.00	0.00	0.00	0.00	0.00	0.00	0.00	0.00	0.00	0.00
C15H30O	0.00	0.00	44.85	0.00	0.00	0.00	0.00	26.59	6.56	0.00
C18H36O	0.00	0.00	0.00	0.00	0.00	0.00	0.00	0.00	3.21	0.00
C18H35Cl	0.00	0.00	0.00	37.50	0.00	0.00	0.00	0.00	0.00	0.00
C22H40O2	0.00	0.00	0.00	16.59	0.00	0.00	0.00	0.00	0.00	0.00
C20H40O	22.93	0.00	48.42	62.27	0.00	20.80	16.36	49.14	31.59	18.95
C16H30O2	0.00	0.00	0.00	0.00	0.00	0.00	0.00	0.00	22.40	0.00
C16H26O2	0.00	0.00	0.00	0.00	0.00	0.00	0.00	34.09	0.00	0.00
C19H26O2	0.00	0.00	0.00	95.62	0.00	0.00	0.00	0.00	0.00	26.34
C6H12O5	0.00	0.00	0.00	0.00	0.00	0.00	0.00	0.00	0.00	0.00
C25H50O	0.00	0.00	0.00	0.00	0.00	0.00	0.00	0.00	0.00	8.68
C21H44O3S	0.00	0.00	0.00	0.00	0.00	0.00	10.79	0.00	0.00	0.00
C26H52	0.00	0.00	0.00	0.00	0.00	13.22	0.00	0.00	0.00	0.00
C22H44	0.00	0.00	0.00	0.00	0.00	0.00	12.08	0.00	0.00	0.00
C19H38O3	0.00	0.00	0.00	0.00	0.00	0.00	0.00	0.00	0.00	0.00
C28H56O2	0.00	0.00	0.00	0.00	0.00	0.00	0.00	0.00	7.72	0.00
C27H48O4	0.00	0.00	8.82	0.00	0.00	0.00	0.00	0.00	0.00	0.00
C19H38O	0.00	0.00	0.00	0.00	0.00	9.09	0.00	0.00	0.00	0.00
C18H36O	0.00	0.00	0.00	0.00	0.00	0.00	0.00	37.54	0.00	0.00
C18H30O2	0.00	0.00	0.00	0.00	0.00	0.00	0.00	0.00	0.00	0.00
C20H40O2	0.00	0.00	7.14	12.63	0.00	0.00	0.00	0.00	0.00	0.00
C18H30O2	0.00	0.00	0.00	0.00	0.00	0.00	0.00	0.00	0.00	0.00
C32H52O2	0.00	0.00	151.79	60.33	0.00	0.00	0.00	0.00	0.00	0.00
	821.18	837.27	1044.02	5931.41	275.27	######	869.94	2086.02	1160.37	2167.11

S11	S12	S13	S14
0.00	0.00	0.00	0.00
0.00	0.00	0.00	78.10
0.00	0.00	0.00	0.00
0.00	0.00	60.62	141.07
0.00	0.00	0.00	0.00
0.00	0.00	0.00	0.00
0.00	0.00	0.00	0.00
28.40	0.00	0.00	0.00
13.62	0.00	0.00	0.00
0.00	0.00	0.00	3.24
32.06	0.00	0.00	0.00
31.33	0.00	0.00	0.00
0.00	0.00	0.00	15.87
0.00	0.00	0.00	0.00
0.00	0.00	0.00	0.00
0.00	19.47	0.00	0.00
116.77	0.00	24.91	64.70
15.17	9.85	0.00	12.19
0.00	0.00	0.00	0.00
0.00	0.00	0.00	0.00
8.98	0.00	0.00	7.47
0.00	0.00	0.00	0.00
8.26	0.00	0.00	4.62
0.00	0.00	0.00	0.00
2.06	0.00	0.00	0.00
0.00	0.00	0.00	0.00
0.00	0.00	0.00	13.61
0.00	0.00	0.00	0.00
75.57	80.42	54.93	129.16
0.00	0.00	0.00	0.00
0.00	0.00	0.00	0.00
0.00	0.00	0.00	0.00
0.00	0.00	0.00	0.00
0.00	0.00	0.00	0.00
10.27	0.00	0.00	11.06
0.00	0.00	0.00	0.00
0.00	0.00	0.00	0.00
18.39	0.00	0.00	0.00
0.00	0.00	0.00	0.00
0.00	0.00	0.00	0.00
48.74	56.37	0.00	149.87
0.00	0.00	0.00	0.00
0.00	13.51	0.00	0.00
0.00	0.00	0.00	34.46
0.00	0.00	0.00	0.00
0.00	0.00	0.00	0.00
0.00	0.00	0.00	34.48
552.64	0.00	283.73	531.48
80.23	0.00	63.70	149.23
0.00	0.00	0.00	0.00
0.00	0.00	0.00	30.81
16.01	0.00	0.00	0.00

0.00	0.00	0.00	0.00
0.00	0.00	0.00	0.00
0.00	0.00	0.00	0.00
0.00	0.00	22.55	35.00
0.00	0.00	0.00	12.65
0.00	0.00	0.00	23.87
0.00	0.00	0.00	0.00
0.00	0.00	0.00	0.00
0.00	0.00	0.00	0.00
0.00	0.00	0.00	0.00
0.00	0.00	0.00	0.00
0.00	0.00	0.00	0.00
0.00	0.00	0.00	44.25
0.00	0.00	0.00	0.00
0.00	0.00	0.00	0.00
0.00	0.00	0.00	0.00
0.00	0.00	0.00	11.33
0.00	0.00	0.00	0.00
0.00	0.00	0.00	50.60
0.00	0.00	0.00	0.00
0.00	0.00	0.00	0.00
0.00	0.00	0.00	0.00
0.00	0.00	0.00	0.00
0.00	0.00	0.00	0.00
0.00	0.00	0.00	0.00
0.00	0.00	16.81	0.00
0.00	0.00	0.00	35.98
0.00	0.00	0.00	0.00
0.00	0.00	0.00	0.00
0.00	0.00	0.00	0.00
0.00	0.00	0.00	0.00
8.99	0.00	0.00	0.00
0.00	0.00	0.00	0.00
0.00	0.00	0.00	0.00
0.00	0.00	0.00	0.00
0.00	0.00	0.00	61.12
0.00	0.00	0.00	0.00
0.00	0.00	0.00	0.00
0.00	0.00	0.00	30.11
0.00	0.00	0.00	0.00
0.00	0.00	0.00	0.00
0.00	0.00	0.00	0.00
0.00	0.00	0.00	0.00
0.00	0.00	0.00	15.90
17.31	0.00	0.00	0.00
0.00	0.00	0.00	22.14
0.00	0.00	21.73	82.65
0.00	0.00	0.00	0.00
0.00	0.00	0.00	0.00
0.00	0.00	0.00	0.00
0.00	0.00	0.00	110.74
0.00	0.00	0.00	0.00
0.00	0.00	0.00	0.00
0.00	0.00	0.00	0.00
0.00	0.00	0.00	0.00
0.00	24.06	0.00	0.00
29.96	0.00	0.00	0.00

0.00	0.00	0.00	13.04
0.00	0.00	184.79	476.03
0.00	38.17	0.00	0.00
0.00	0.00	0.00	0.00
0.00	0.00	0.00	0.00
0.00	0.00	0.00	0.00
0.00	0.00	0.00	0.00
0.00	21.17	0.00	0.00
0.00	0.00	0.00	0.00
0.00	0.00	0.00	0.00
0.00	0.00	0.00	0.00
0.00	25.40	0.00	0.00
0.00	33.15	0.00	0.00
0.00	20.93	0.00	0.00
11.69	0.00	0.00	0.00
0.00	0.00	0.00	0.00
0.00	0.00	0.00	0.00
0.00	0.00	0.00	0.00
0.00	0.00	0.00	0.00
0.00	0.00	0.00	0.00
0.00	0.00	0.00	66.57
0.00	0.00	0.00	0.00
0.00	0.00	0.00	0.00
0.00	13.59	31.13	0.00
0.00	13.75	0.00	0.00
0.00	0.00	0.00	0.00
0.00	0.00	0.00	0.00
0.00	0.00	0.00	0.00
0.00	0.00	0.00	0.00
0.00	0.00	0.00	0.00
0.00	0.00	0.00	0.00
0.00	0.00	0.00	0.00
0.00	0.00	0.00	0.00
0.00	0.00	0.00	0.00
0.00	0.00	0.00	0.00
23.12	0.00	27.35	0.00
32.98	47.13	0.00	0.00
0.00	0.00	0.00	0.00
0.00	0.00	0.00	0.00
0.00	38.24	0.00	0.00
32.11	0.00	26.50	0.00
0.00	0.00	0.00	46.41
0.00	0.00	0.00	0.00
0.00	0.00	0.00	0.00
0.00	0.00	0.00	0.00
0.00	0.00	0.00	0.00
0.00	11.47	0.00	0.00
26.11	0.00	0.00	71.47
0.00	0.00	0.00	0.00
15.12	0.00	0.00	0.00
0.00	11.51	0.00	0.00
0.00	0.00	0.00	0.00
0.00	0.00	0.00	0.00
0.00	0.00	0.00	0.00
0.00	0.00	0.00	0.00
0.00	0.00	0.00	0.00
0.00	0.00	0.00	0.00

0.00	0.00	0.00	0.00
0.00	0.00	0.00	0.00
0.00	0.00	0.00	9.96
4.97	11.96	0.00	0.00
0.00	0.00	0.00	0.00
0.00	0.00	0.00	0.00
0.00	0.00	0.00	0.00
0.00	0.00	0.00	0.00
0.00	19.03	0.00	0.00
0.00	23.44	0.00	0.00
0.00	0.00	0.00	0.00
0.00	0.00	0.00	0.00
0.00	0.00	0.00	0.00
0.00	0.00	0.00	0.00
7.40	0.00	0.00	0.00
0.00	0.00	0.00	11.78
0.00	0.00	0.00	0.00
0.00	0.00	0.00	0.00
0.00	10.75	0.00	0.00
0.00	0.00	0.00	0.00
0.00	0.00	0.00	0.00
0.00	0.00	0.00	0.00
0.00	0.00	0.00	0.00
0.00	0.00	0.00	0.00
0.00	0.00	26.11	0.00
0.00	3.56	0.00	8.78
0.00	0.00	0.00	0.00
0.00	0.00	0.00	0.00
0.00	0.00	0.00	0.00
0.00	0.00	0.00	19.34
0.00	0.00	0.00	0.00
0.00	0.00	0.00	0.00
0.00	0.00	0.00	0.00
0.00	0.00	6.88	0.00
0.00	0.00	0.00	0.00
0.00	0.00	0.00	0.00
0.00	0.00	0.00	0.00
0.00	0.00	0.00	0.00
12.38	0.00	0.00	0.00
0.00	0.00	18.36	0.00
0.00	0.00	0.00	0.00
0.00	0.00	0.00	0.00
0.00	0.00	0.00	0.00
0.00	0.00	40.22	0.00
0.00	0.00	0.00	0.00
0.00	0.00	9.77	25.04
0.00	0.00	0.00	0.00
1280.64	546.93	920.07	2696.20
